# Quantitative X-ray phase-contrast digital histology of liver metastases in a mouse model

**DOI:** 10.1038/s41598-025-96049-9

**Published:** 2025-05-26

**Authors:** Lorenzo Massimi, Sara Vitale, Laura Maugeri, Luca Businaro, Giuseppe Gigli, Micol E. Fiori, Alessia Cedola

**Affiliations:** 1 CNR-Nanotec (Institute of Nanotechnology), Rome, Italy; 2 Department of Oncology and Molecular Medicine (OMM), Istituto Superiore di Sanità, Rome, Italy; 3 CNR-IFN (Institute of Photonics and Nanotechnologies), Rome, Italy

**Keywords:** Cancer imaging, X-ray tomography

## Abstract

Investigating the tissue modifications occurring as a consequence of tumour development is an important goal in preclinical medical research, as it can provide a better understanding of the mechanisms behind its origin and spread. Tumor microenvironment has a supportive role in cancer development and can be exploited as a therapeutic target to prevent and contrast metastatic spread, which usually leads to a poor prognosis. In this work, a colorectal cancer model of liver metastasis is used to perform proof-of-concept quantitative investigations of the changes occurring in murine liver tissue due to the formation of metastases. X-ray phase contrast imaging performed with synchrotron radiation was used to obtain high resolution and contrast on soft tissues with minimum sample preparation and a large field of view on a 3D volume. A pixel size of 3 µm, and 0.7 µm have been used. to visualize and quantify liver microvasculature, referred to as sinusoids, and to identify significant morphological differences between control and metastatic tissues. A reorganization of the hepatic tissue, characterized by increased vascularization around the metastatic lesions coupled with a significant reduction in the sinusoidal network in the distal liver parenchyma was observed. X-ray findings are also supported by conventional histology, proving X-ray phase contrast imaging as an informative complementary technique.

## Introduction

The term metastasis describes the migration of cancer cells from the original anatomical site to a distant organ where they can form a new tumour mass. The presence of metastasis is a critical element in tumour staging, known as TNM (Tumour-Node-Metastasis), which also requires the assessment of the size of the tumour and the involvement of lymph nodes. Unfortunately, the presence of metastases places the tumour into a stage IV for which the efficacy of treatments is significantly reduced^1^. Colorectal cancer (CRC) is one of the three most common cancers worldwide and the fourth leading cause of cancer-related deaths^2^. Even after curative resection of the primary tumour, around 30-40% of CRC patients will develop metastases in the subsequent years^3^. The liver is the most common organ of distant metastasis in CRC, due to its anatomical and microenvironmental characteristics^4^. In this framework, preclinical investigations are still important in assessing the mechanisms behind the formation of metastases. Among all the tools available to medical research, including conventional histology and volumetric confocal microscopy, X-ray phase contrast imaging is a fundamental technique providing structural information that is difficult or impossible to achieve otherwise. It combines the high penetration depth as conventional X-ray imaging, but with the advantage of improved contrast on soft tissues and minimal sample preparation as already shown in other pathological conditions^5–7^. While it can also be performed in the laboratory using optical elements^8,9^, when the free-space propagation technique is used with synchrotron radiation, it also provides a high sample throughput in addition to high image contrast and resolution, allowing the scan of a sample in a time ranging from a few minutes to dozens of minutes, depending on the experimental setup. In the present work, we used the capability of X-ray phase contrast tomography of providing images of soft tissues with high resolution and contrast to present extensive volumetric analyses involving the blood vessels as well as the tissue itself in a mouse model of liver metastases. We found significant modifications with a massive reduction in the micro-vascularization and a change in the tissue appearance. Both these aspects have been quantified using appropriate estimators, finding statistically relevant alterations between the control and metastatic cases. It is worth noting that the low number of investigated specimens keeps our results at a proof-of-concept level. However, additional histological analyses have been used to support our findings. In addition, investigating a chemotherapy-treated sample suggested that these approaches may also serve in future studies to assess the effect of drug treatments on the tumor microenvironment.

## Methods

### Sample preparation

#### Isolation of colorectal liver metastasis stem cells

Surgical specimens of colorectal liver metastasis were obtained from patients undergoing hepatectomy with curative intent at the University Polyclinic A. Gemelli, in agreement with the standards of the institutional Ethics Committee on human experimentation, authorization n. CE5ISS 09/282 and in accordance with all the relevant guidelines and regulations. Informed consent was obtained from all the patients for the use of their tissue in research. Samples were processed as previously described^10^. The cells derived from these specimens have been subsequently used for splenic injection in mice.

#### Animals

Mice were maintained in specific pathogen-free standard housing conditions (20 ± 2 $^{\circ }$C, 50 ± 5% humidity, 12h–12h light-dark cycle, with food and water ad libitum). All in-vivo experimentations complied with the EU Directive 63/2010 and were included in an experimental protocol approved by the Institutional Animal Experimentation Committee at the Istituto Superiore di Sanità (Rome) and the Italian Ministry of Health (N. 1157/2020-PR). Six to seven week-old male NOD.Cg-Prkdcscid Il2rgtm1Wjl/SzJ (NSG) mice were from Charles River, housed in the animal facility at the Istituto Superiore di Sanità and used after a 7-day acclimatization period. All experiments followed the Guidelines for the Care and Use of Laboratory Animals, including ARRIVE guidelines^11^. Four animals per condition, i.e. healthy and metastatic, have been considered in this work. One animal per condition was used for the X-ray investigation, while all of them have been used for histological CD31 and H&E staining to support the X-ray findings.

#### Liver metastases

Patient-derived CML-SCs stably transduced to express luciferase and GFP were injected in the spleen of NSG mice. For splenic injection, the mice were anesthetized with Ketamine (100 mg/kg) and Xilazine (10 mg/kg) given IP before the surgical procedure as described in (13). Tumor-injected mice were divided into two groups (4 mice/group): untreated (metastatic group) and 5FU+Oxalipatin (FOX)-treated (chemotherapy group). A group of four mice was also included as healthy control. Liver metastasis formation was monitored weekly measuring in vivo bioluminescence (BLI) with an IVIS imaging system (Perkin Elmer). Before imaging, mice were injected with 150 mg/kg luciferin IP. Mice were sacrificed on day 27 after spleen injection and livers were excised and collected.

### X-ray phase contrast data acquisition

X-ray phase contrast tomography datasets have been acquired on the multiscale setup available on the ID17 beamline at the European Synchrotron Radiation Facility (ESRF) in Grenoble^12^. Measurements were performed in polychromatic (pink) beam mode with a propagation distance of about 20 cm. Pink beam features a mean energy of about 30 keV^12^. The image was detected with an indirect conversion system based on a PCO edge 5.5 sCMOS camera coupled with magnifying optics focused on a scintillator screen. The effective pixel size in use for the experiment was 3.1 µm (low-resolution scans) and 0.7 µm (high-resolution scans), resulting in a field of view of about $7.9\times 6.7$ mm$^2$ and $1.8\times 1.5$ mm$^2$, respectively. The tomography was performed in both cases in the extended field of view mode, acquiring 3600 projections over 360$^{\circ }$. In the extended field of view mode, the axis of rotation is placed close to the edge of the detector field of view instead of at the centre. Therefore, a 360$^{\circ }$ rotation allows for the reconstruction of an image larger than the actual detector size. The increase in fov depends on the position of the rotation axis. The increase was about 1.8 times the actual detector size for the scans presented in this work. During the acquisition, samples were kept in paraffin blocks glued on the sample stage and subsequently used for histological slicing. Tomography reconstruction has been performed with PyHST2^13^. For phase retrieval the Paganin filter was used on the acquired projections with a ratio $\delta /\beta = 500$^14^. Ring artefact reduction has been made on the polar transformed reconstructed slice using Fourier frequency filtering^15^.

### Histology

Metastatic or healthy livers were fixed in neutral buffered (10%) formalin solution and paraffin-embedded (FFPE: Formaline Fixed Paraffine Embedded). Tissue sections were stained for Hematoxylin and Eosin (H&E) (Bio-Optica) or CD31 immunohistochemical (IHC) (clone C31/2876R, Abcam) using Ultra Tek HRP Anti-Polyvalent (AEC) Staining System (Scy Tek Laboratories). Images (40$\times$) were obtained with Vectra Polaris$^{TM}$ Automated Quantitative Pathology Imaging System (Akoya Biosciences) and analyzed with QuPath software and ImageJ.

### Data processing

Data analysis was performed using custom-designed scripts in Matlab and ImageJ. The first step involved the segmentation of blood vessels. Due to the presence of both large and small vessels, a combined approach has been used. Small blood vessels have been extracted using Frangi’s vesselness function^16^ with the following parameters: scale 1–10, $\alpha =\beta =0.5$, C = 0.013, and selecting all the voxels with probability P > 0.16. The parameters $\alpha , \beta$ and C control the filter’s sensitivity. Specifically, $\alpha$ and $\beta$ control the deviation of the eigenvalues of the ellipsoid from a blob-like structure, allowing to distinguish between a plate-like structure and a line-like structure. The parameter C controls the deviation of the intensity of the analysed region from the background^16^. The larger vessels have been segmented using an intensity threshold approach. The two binary segmented volumes have been subsequently merged. Large vessels have been excluded from the analyses since they are not expected to be affected as the smaller ones. The threshold between large and small vessels has been empirically set to a diameter of 15 µm. This diameter was chosen to be sure to include all the sinusoids vessels in the calculation. The in-plane root-mean-square (RMS) of the grey values within the tissue has been calculated on whole low-resolution CT scans on a single slice basis to quantitatively describe the observed differences in tissue morphology using a squared sliding window of 30 pixels per side. Pixels belonging to blood vessels have been excluded from the calculation, and the average value of each window has been subtracted before the calculation. We also introduced the vascular volume fraction (VVF) to quantify variations in the vascular network. VVF was calculated on a volumetric basis on the high resolution CT scans. The VVF was defined as the ratio between the number of voxels belonging to the blood vessels and those belonging to the tissue, calculated on a large, manually selected volume of interest or a sliding cubic window of 50 pixels per side on the binary segmented volume. In addition, a radial VVF has also been calculated for the region surrounding the metastases and defined as the VVF within a shell of increasing distance around the manually segmented boundary of the metastases. The result for each shell is plotted against the major axis of the best fit ellipsoid. Additional quantities have also been calculated. Precisely, the vessel lengths have been computed via curved interpolation of the voxels belonging to each branch as obtained from the skeletonization of the segmented binary volumes. A branch is defined according to the ImageJ skeleton classification. The nearest neighbour distance between blood vessels has been calculated for each vessel as the minimum distance between their centre of mass, defined along a curved reference system so that it lies within the blood vessel itself. The level of vascularization has also been quantified on the histological CD31-stained images by segmenting the colour range associated with the staining and expressing the result as a 2D volume fraction, defined as the ratio, expressed as a percentage, between the pixels belonging to vessels and the number of pixels in the image. For the CD31 analysis about 40 regions of interest per animal have been considered, covering 0.25 mm$^2$ in total.

## Results

**Fig. 1:**
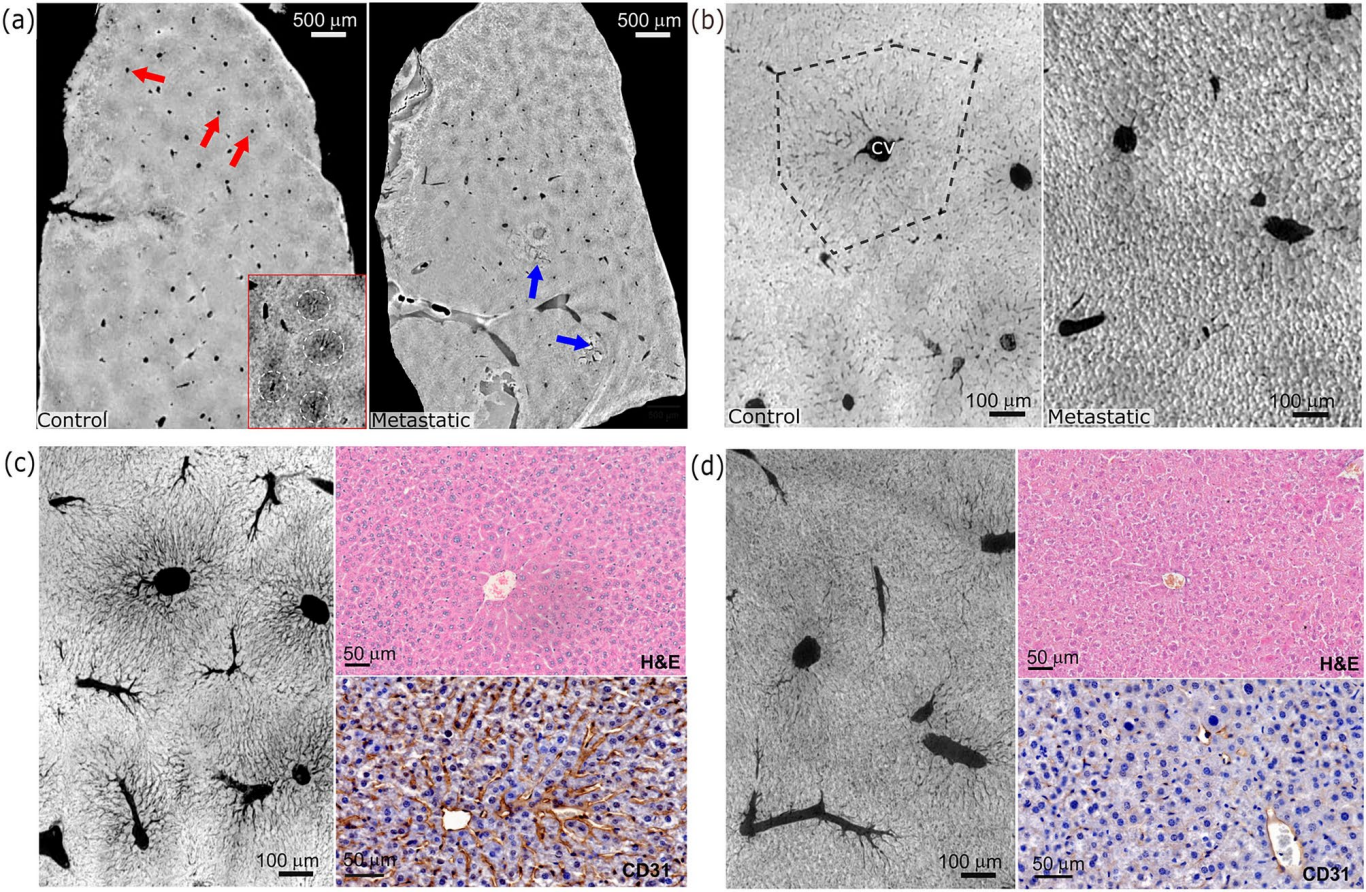
Panel (**a**) and (**b**) show low and high resolution X-ray CT slices of FFPE liver specimens of control and metastatic mice, respectively. Red and blue arrows point at the central veins and metastatic nodules, respectively. Panel (**c**) and (**d**) show the minimum intensity projection over 50 µm of the same regions reported in panel (**b**) for both the conditions combined with H&E and CD31 immunohistochemical staining.

FFPE tissues were analysed by X-Ray phase contrast CT, comparing healthy metastatic and chemotherapy treated livers. Scan results at different spatial resolutions and histological H&E and CD31 sections for both cases, i.e. control and metastatic, are reported in Fig.1.

The low-resolution scan of the control specimen shows several almost circular blood vessels with a diameter above 15 $\mu$m compatible with central veins forming the liver functional units (the lobules), as shown in Fig. 1a (left image). These blood vessels are surrounded by lower-density regions regularly spread across the sample, giving the tissue a patchy-like appearance. This is indicated by red arrows in Fig. 1a, and it is better highlighted in a contrast stretched image reported in the bottom-right inset. The high-resolution CT scan for the control specimen reported in Fig. 1b shows a detailed magnification of one of these functional units. Specifically, a structure compatible with the organisation of the liver lobule, formed by a central vein usually surrounded by six units, comprising a portal vein, a hepatic artery and a bile duct (portal triad), can be identified (see black dashed line). In addition, small blood vessels exhibiting a diameter around 3 µm, as reported in the diameter distribution in the supplementary information Fig. S1, are found. The minimum intensity projection of the same region in Fig. 1c reveals how these vessels form a dense capillary bed mainly spreading from the central vein. This microvasculature is compatible with the sinusoids, in agreement with previous findings^17^, and the CD31 histological staining validates the assignment of these features to vessels. The CD31 is a specific endothelial marker staining small and large vessels (DAB staining). From a morphological point of view, the tissue appears homogeneous, with hepatocytes barely distinguishable. A similar appearance is also found in the H&E stained histological section. The analyses on the metastatic liver show how the presence of cancerous lesions has a substantial impact on liver parenchyma organization. The low-resolution image, reported in Fig. 1a, shows a local disruption of the tissue arrangement around the metastatic nodules (see blue arrows). The high-resolution image in Fig. 1b also reveals a severe alteration at the microvascular level. The tissue appears dense, with well defined cellular edges and a poor sinusoidal network. This is well visible in the minimum intensity projection in Fig. 1d, which confirms a significantly reduced capillary bed. These features can also be observed by H&E and CD31 stainings. It is worth noting that the histological sections do not show the same regions reported in the X-ray images but are similarly focused on a central vein to allow a qualitative comparison.

**Fig. 2:**
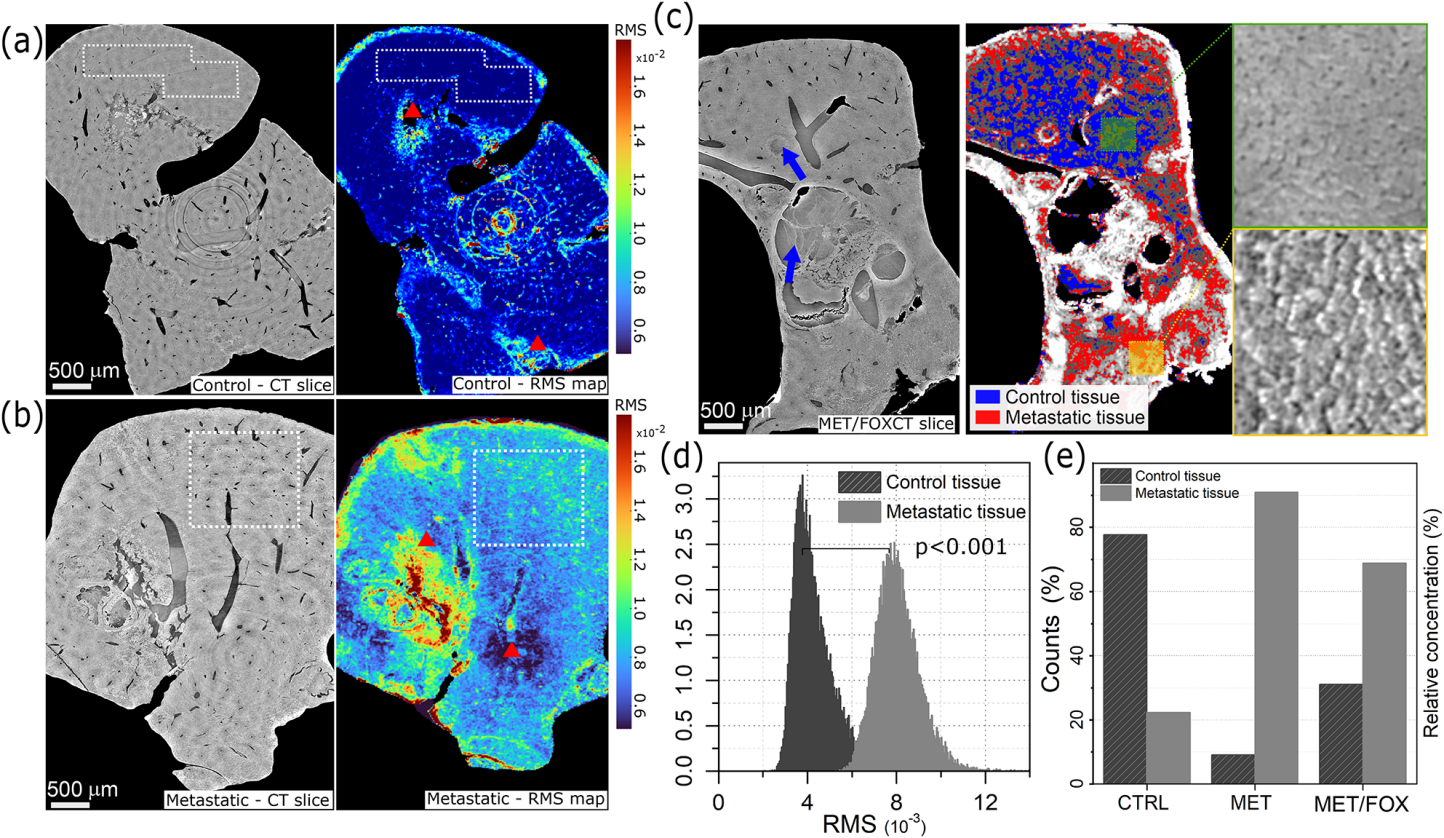
Panels (**a**) and (**b**) show a tomography slice (left side) and the corresponding RMS map (right side) for the control and metastatic specimens, respectively. Red triangles point at high RMS values due to damaged tissue or processing artifacts. Panel (**c**) shows a low-resolution CT slice of a chemotherapy-treated sample (left) and the result of tissue classification based on its RMS value (right), combined with high-resolution crops in the zoomed-in the rightmost panels. Blue arrows point at metastatic nodules. Graphs in panels (**d**) and (**e**) depict the distribution of RMS values for both healthy and metastatic tissue. These values were extracted from regions highlighted with white dashed lines in panels (**a**) and (**b**) and the overall distribution across the two types of tissues for control (CTRL), metastatic (Met) and chemotherapy-treated (Met/FOX) samples.

The qualitative differences detected in histological morphology can be exploited to quantitatively distinguish healthy and metastatic tissues based on X-ray phase contrast images as illustrated in Fig. 2.

Specifically, the apparent difference in roughness described by the root-mean-square (RMS) has been leveraged. An RMS map was calculated using a sliding window for all the specimens on the low-resolution CT slices. The result is displayed as colour maps on the right of Fig. 2a and b for the control and the metastatic samples, respectively. The metastatic tissue exhibits a higher RMS ranging from $8\cdot 10^{-3}$ to $12\cdot 10^{-3}$ while the control remains below $6\cdot 10^{-3}$. The difference is statistically significant with p<0.001, as shown by the RMS distribution plot in Fig. 2d. Regions with markedly high or low RMS values, indicated by red triangles in Fig. 2a and b, can be observed due to metastatic nodules, sample damage or CT artefacts such as originated by ring suppression or residual rings. Consequently, only values from intact regions, highlighted by dashed white rectangles in Fig. 2a and b, were considered in the RMS distribution. An additional specimen from a mouse that received chemotherapy treatment was also included to explore the potential of this approach in classifying unknown sample conditions. A representative slice is reported in Fig. 2c. The tissue in this slice has been classified as healthy or metastatic according to its RMS value. Specifically, a tissue region is assigned to the healthy or metastatic class if its RMS value falls within the corresponding mean value plus or minus 1.5 times the standard deviation. Metastatic nodules or blood vessels were excluded from the classification. The result is shown in the right image of Fig. 2c. In the upper region of the slice, a large area exhibits an RMS value consistent with healthy tissue, supported by a high-resolution CT image in the zoomed-in inset (Fig. 2c), right panels). Similarly, in the lower part of the specimen, the RMS value indicates the presence of metastatic tissue, which is also confirmed by the CT image. This classification method was applied across all the slices from the three specimens. The results are reported in Fig. 2e as a relative percentage, considering only classified pixels. It is confirmed that the control and the metastatic specimens have a significant prevalence of healthy and metastatic tissues, respectively, as expected. On the other hand, the classification of the tissues in the chemotherapy-treated sample reveals a higher proportion of healthy tissue compared to the metastatic sample, indicating an intermediate situation between the control and metastatic specimens. It is worth mentioning that also the control specimens present a non-zero amount of voxels classified as metastatic tissue. This effect may arise from the classification approach since the single-slice RMS distributions from both conditions partially overlap. In addition, misclassified CT artefacts, such as not completely removed rings or damaged areas of the specimen, may contribute to misclassified regions.

**Fig. 3:**
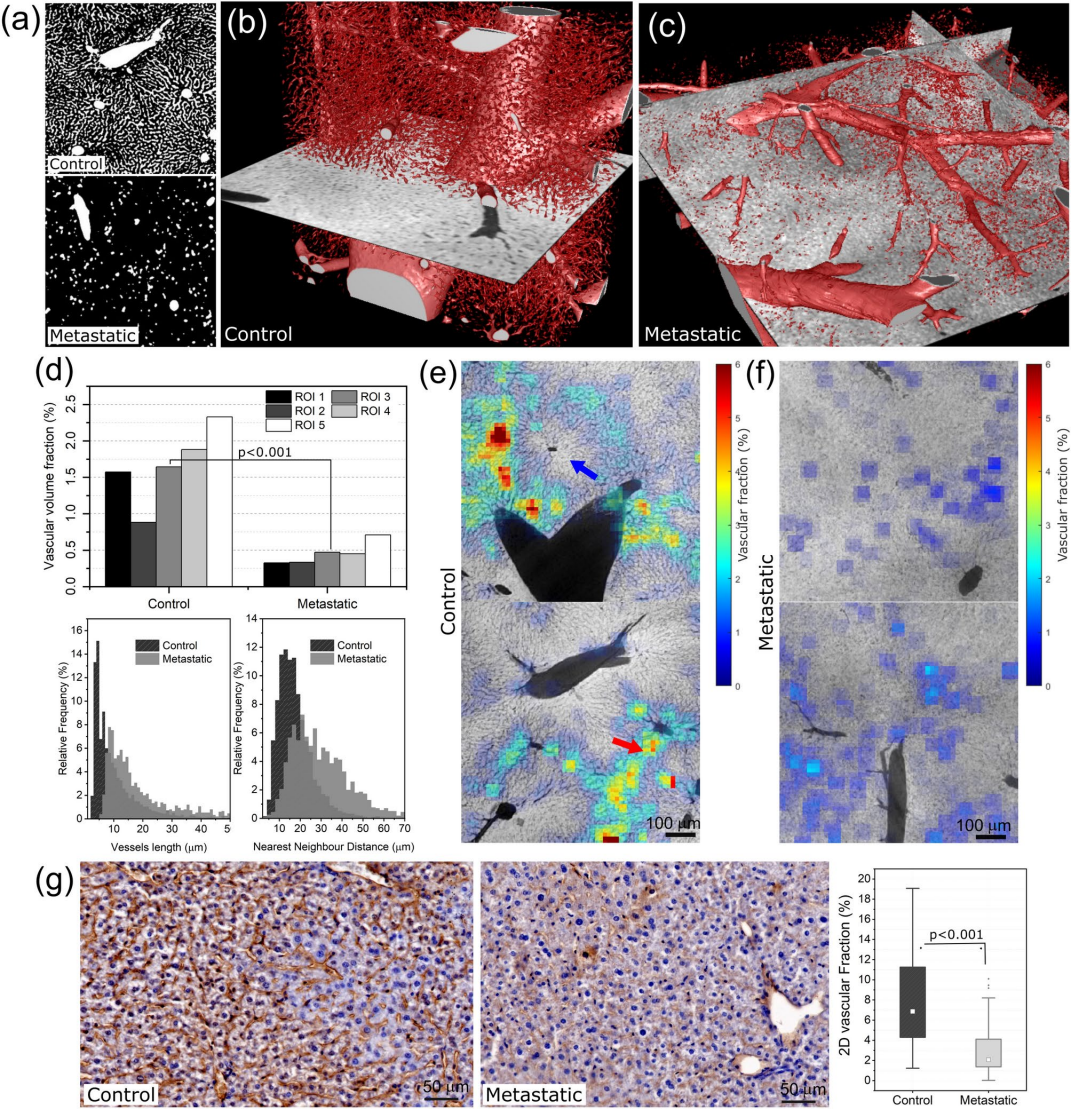
Panel (**a**) shows a minimum intensity projection over 50 $\mu$m of the segmented blood vessels for both the control (top) and metastatic (bottom) cases. Panels (**b**) and (**c**) show a 3D rendering of the segmented vascularization in the control and metastatic cases, respectively. Panels (**d**) show the quantitative plots for the VVF calculated on five different ROIs, the vessel length and the nearest neighbour distance for both the control and metastatic specimen. For the calculation of the VVF, vessels larger than 10 µm have been excluded. Panels (**e**) and (**f**) show a spatial map of the VVF as a colour map, overlapped with the minimum intensity projection of the CT slices across about 10 µm for the control and metastatic mouse, respectively. Red and blue arrows point at high and low density regions, respectively. Panel (**g**) shows an example of two histological slices stained with anti-CD31 antibody for both the control and the metastatic case. The result for all the animals is summarized in the box plot.

The previous analyses clearly indicated a significant influence of the tumor on the liver microvasculature. This aspect was further investigated from a quantitative point of view, and the results are shown in Fig. 3.

The result of segmentation is shown for both the samples as minimum intensity projection of the binary volume in Fig. 3a, clearly revealing the differences in the sinusoids size, amount and distribution between the two samples. This is further supported by the 3D rendering of the whole segmented volumes in Fig. 3b and c for the control and metastatic specimens, respectively. To quantitatively support these qualitative observations, the VVF has been calculated in five randomly selected regions of interest (ROIs) of equal size (about $5\cdot 10^{-3}$ mm$^3$) and reported in the top graph of Fig. 3d. The graph shows that in all the investigated ROIs, the VVF is significantly higher (p<0.001) for the control (mean value 1.65%) compared to the metastatic specimen (mean value 0.45%). Additional quantities have been computed to further characterize the capillary bed modification, i.e., the vessel’s length and the nearest neighbour distance. Both distributions have been normalized to the total number of vessels in each specimen. The length distribution clearly shows an increase in the average vessels’ length in the metastatic specimen. Specifically, in the control specimen the peak is around 5 µm, whereas in the metastatic sample it shifts to above 10 µm. In addition, the nearest neighbour distance distribution also moves from the weighted average value of 17.8 µm in the control specimen to 32.5 µm in the metastatic one. The increase in the vessel length found in the metastatic case can be explained by considering that the density of the sinusoid network decreases. Therefore, connections are formed between vessels further away from each other. On the other hand, in the control case, the capillary bed has a higher density, so vessels are closer, resulting in shorter branches. This is consistent with the increased minimum distance between adjacent vessels in the metastatic case. In Fig. 3d, a variation between the VVF values estimated in different ROIs can reach up to about 70%, indicating significant local variability in VVF. To further elucidate this point, the VVF has been calculated on a sliding cubic window of size 50 $\times$ 50 $\times$ 50 voxels and overlapped with the minimum intensity projection of the corresponding tomographic slices using the VVF map normalized between 0 and 1 as opacity map, as shown in Fig. 3e, f for the control and the metastatic specimens, respectively. The presence of local variations in the VVF is evident. In the control case, VVF values range from as low as 1% up to about 6%. Interestingly, vessels either surrounded by a low (below 1%) or high (up to 6%) density of capillaries can be observed, as indicated by blue or red arrows in the top and bottom panels of Fig. 3e. This observation agrees with the pattern of vessel density increasing towards a central vein, as depicted in the histological staining in Fig. 1b. On the other hand, in the metastatic case, a more dispersed distribution can be found with values that do not exceed 3%. CD31 staining has been performed on additional samples to support the X-ray findings, and the results are presented in Fig. 3g. In the control liver, the network of the sinusoids appears uniformly distributed and dense, while it appears greatly reduced in the metastatic tissue. A 2D vascular fraction was calculated on the CD31 stained images by segmenting the blood vessels in different ROIs obtained from all the specimens. The results are shown as a box plot on the right panel of Fig. 3g, confirming that the metastatic case has a significant (p < 0.001) lower 2D vascular fraction compared to the control. 

**Fig. 4:**
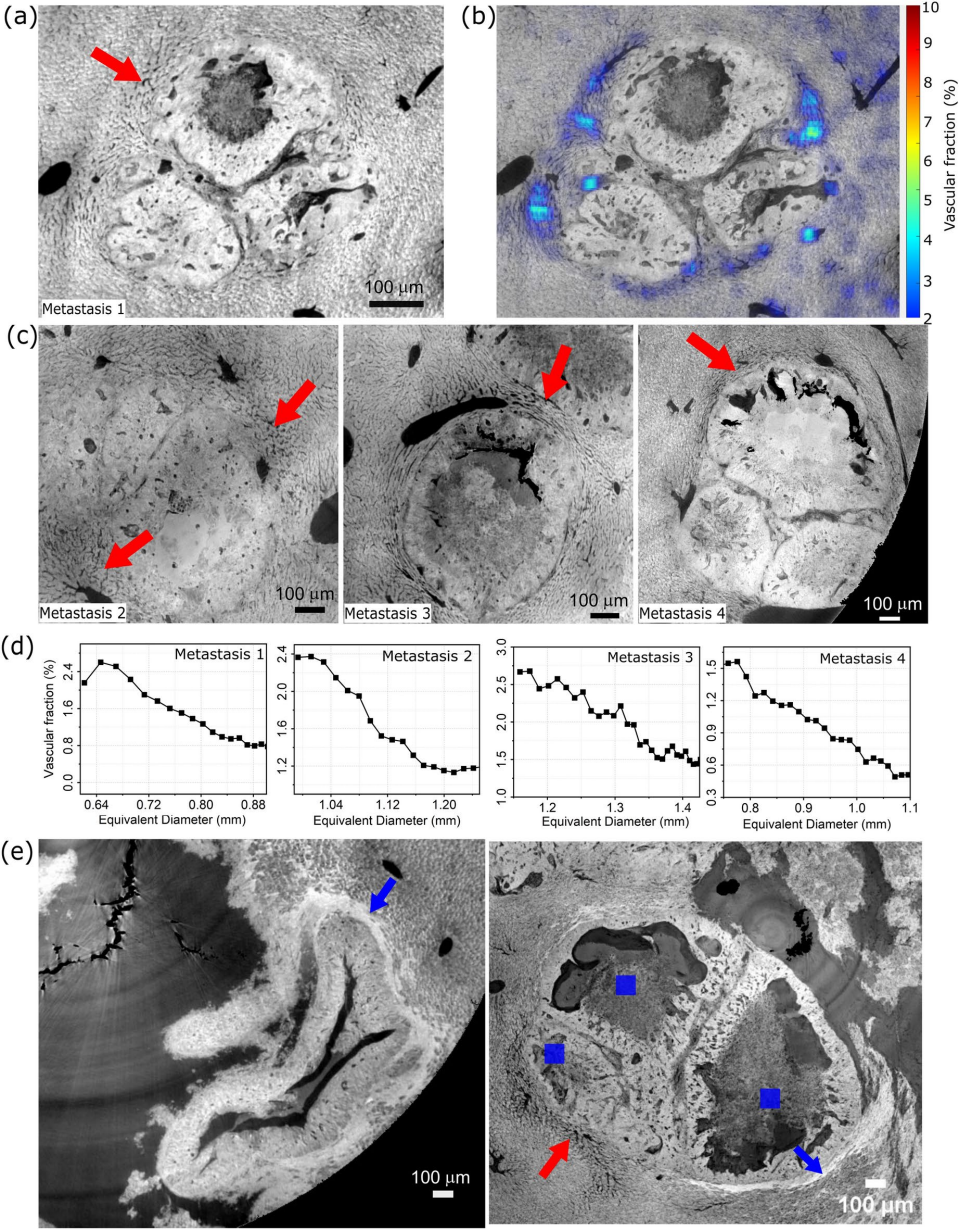
Panels (**a**) and (**b**) show a minimum intensity projection of a liver metastasis over about 20 $\mu$m, and the same image overlapped with a radial VVF colour map, respectively. Panel (**c**) reports the minimum intensity projection for other metastatic nodules with the plot of their radial vascular volume fraction (including the one shown in panel (**a**)) in panel (**d**). Panel (**e**) shows different metastases exhibiting a high-density rim (pointed by blue arrows) compatible with a desmoplastic shell. In all the panels red arrows point at a density-increased capillary bed, and blue squares mark necrotic tissue.

So far, the results show a severe alteration of the liver parenchyma, with a significant capillary reduction in the presence of metastatic lesions. When analyzing the tissue surrounding the tumor, we observed instead a dense distribution of blood vessels and capillaries, consistent with expected tumor-induced angiogenesis required for nutrient supply supporting the rapidly growing cells. Detailed analyses of the capillaries in the metastatic tissue are reported in Fig. 4.

In Fig. 4a, the minimum intensity projection of a metastasis is shown as an example. The metastatic nodule is severely disrupting the local tissue arrangement, and it appears to be surrounded by a dense network of capillaries, resembling the appearance of the healthy tissue, as pointed out by the red arrows in all the panels. This qualitative observation has been quantitatively validated by the VVF colour map overlaid on the CT slice shown in Fig. 4b. The map indicates values up to about 6% around the metastasis, which decreases with increasing distance from it. To better quantify this variation, a radial VVF has been introduced. The results for the metastasis reported in Fig. 4a and for the other three metastatic nodules in Fig. 4c are reported in the graphs in Fig. 4d, where the VVF values are plotted against the central axis of the best-fit ellipsoid of the shell. The VVF starts between 2.5% and 1.5%, which is close to or slightly above the average value of 1.65% observed in the control and decreases almost linearly to about 0.5% consistent with the baseline value of 0.45% found in the metastatic tissue (Fig.3d). In addition, from a qualitative perspective, different metastasis can be recognized. These are shown in Fig. 4(e)w. They exhibit an evident rim separating the metastatic tissue from the surrounding liver tissue (indicated by blue arrows). Remarkably, where this rim is present, the density of the capillary bed is reduced, and more necrotic tissue (blue squares) is present in the surrounding regions. On the other hand, in the region closer to the high-density capillary bed, minimal necrosis can be observed, suggesting that the vessel network is providing enough support for cellular growth.

## Discussion

We presented a comprehensive quantitative analysis of the micro-environment of liver metastases, in a mouse model for advanced colorectal cancer. Our study combines images obtained at various spatial resolutions using X-ray phase-contrast tomography with synchrotron radiation, alongside conventional histology. Healthy liver tissue is organized in morpho-functional units, known as lobules. In the lobule, blood enters via portal veins and the hepatic arteries, located within portal triads, and moves through a dense capillary bed, known as sinusoids, to the central vein located at the center of each lobule^18,19^. Under pathological conditions, changes in sinusoid morphology alter the liver microcirculation and, consequently, its function^20,21^. Due to their complex spatial arrangement, 2D techniques such as histological slicing or confocal microscopy can provide only a limited description of such modifications compared to volumetric techniques. Therefore, X-ray imaging has already been used to quantitatively investigate the 3D arrangement of the sinusoids both in absorption and phase contrast modes. Remarkably, it has been previously reported that X-ray phase-contrast did not provide enough contrast to visualize the sinusoids^17^. Therefore, some previous analyses were based on X-ray absorption contrast using osmium-based stained specimens. Our work confirms that paraffin-embedded liver specimens can provide enough contrast to visualize sinusoids without staining when imaged with free-space propagation X-ray phase contrast^22^. Sinusoids can be easily detected and extracted using standard imaging segmentation algorithms^16^. In addition, we visualize the lobules arrangement that appears as low contrast regions in Fig. 1a. The different tissue contrast may be due to the diverse metabolic activity of the hepatocytes since blood oxygen content decreases moving from the periphery (periportal zone) to the center of the lobule (pericentral zone)^23^. Modifications of the vascular network are generally expected in the presence of a tumour. These changes may affect both the macroscopic circulation, due to the presence of large masses, and the microcirculation, which can be altered to support tumor growth. Modification of the liver circulation has already been reported in other works based on X-ray phase contrast imaging^22^. However, these studies, focused only on the tumour vascularization reporting increased vessels’size and density on its surface. Similarly, we observed remodeling of the capillary network, which appears reduced in the tissue distant from the metastases but increased in a region immediately surrounding them, reaching values similar to or higher than those of the healthy tissue. In addition, our work, for the first time, shows the presence of severe alterations in the microcirculation also in areas of the tissue distant from the tumour lesions which have been not investigated yet. In the presence of liver metastatic lesions, we observed a reduction of the sinusoidal network paired with altered appearance of the distal hepatic tissue. We introduced two estimators to quantify these modifications: the in-plane root-mean-square (RMS) and the vascular volume fraction (VVF). RMS has been calculated on the low-resolution scans, allowing the analysis of the whole specimen. On the other hand, the VVF analysis has been performed on the high-resolution scans, covering only smaller portions of the specimen since blood vessels were barely visible in the low-resolution images. Notably, through RMS values, we were able to classify healthy and metastatic tissues. For this analysis, low-resolution CT slices have been used with the advantage of a large field of view, covering a few centimetres wide samples with a single measurement. Applying the same estimator to a chemotherapy-treated sample revealed a more balanced distribution of both tissues, suggesting that our approach could be effectively used to track the progression and efficiency of cancer treatments. The VVF has been used to describe sinusoid modifications within a volume of interest. VVF has also been calculated on sliding cubes, providing spatially resolved maps that allowed the description of local changes of the sinusoids. The high contrast also allowed us to identify an additional morphological feature, known in the literature as a desmoplastic rim, a fibrotic layer encapsulating the metastatic lobule, consistent with previous findings^24^. Remarkably, in areas where this rim is present, higher levels of necrotic tissue within the nodule and absence of blood vessels are observed, suggesting that the formation of the rim may represent a later stage in the evolution of the metastasis. The reduction in the microvasculature has further been supported by calculating the 2D vascular fraction on histologically CD31 stained sections. It is worth noting that a direct validation of the X-ray findings is unfeasible due to the unique 3D capability of X-ray phase contrast tomography, which cannot be easily replicated with conventional histology. While our work is at a proof-of-concept stage, as we have examined only a single specimen for each condition, it introduces a set of results that may serve as a starting point for future detailed investigations and analyses. These efforts could significantly expand our knowledge of liver oncology and enable exploration into diverse pathological conditions.

## Supplementary Information

Supplementary Information 1.

## Data Availability

The datasets analysed during the current study are available from the corresponding author on reasonable request.
