# Supplementary material for: Quantitative X-ray phase-contrast digital histology of liver metastases in a mouse model
Source: Sci Rep. 2025 May 26;15:18410. doi: 10.1038/s41598-025-96049-9 (PMC12106767; doi:10.1038/s41598-025-96049-9)
Supplement: Supplementary file 1 — Supplementary Information 1. [file 41598_2025_96049_MOESM1_ESM.pdf]

Quantitative X-ray phase-contrast digital histology of liver metastases in a mouse model: supplementary material

Lorenzo Massimi[1], Sara Vitale[2], Laura Maugeri[1], Luca Businaro[3], Giuseppe Gigli[1], Micol E. Fiori[2], and Alessia Cedola[1]

[1]CNR-Nanotec (Institute of Nanotechnology), Rome, Italy
[3]CNR-IFN (Institute of Photonics and Nanotechnologies), Rome, Italy.
[2]Istituto Superiore di Sanità, Department of Oncology and Molecular Medicine (OMM), Rome, Italy

February 20, 2025

Segmentation of smaller blood vessels using Frangi's vesselness function allowed us to estimate the diameter of the sinusoids. The result is presented in Fig.S1 for both the control and metastatic specimen. The calculation was performed on different manually selected volumes of interest, covering about $5 \cdot 10^{-2}$ mm^3 in total, distant from larger vessels. The histogram distributions both exhibit a peak around 3.1 μm and values extending above 4.5 μm. The range of values agrees with the size expected for the sinusoids, and no significant change from the control and the metastatic specimen is observed.

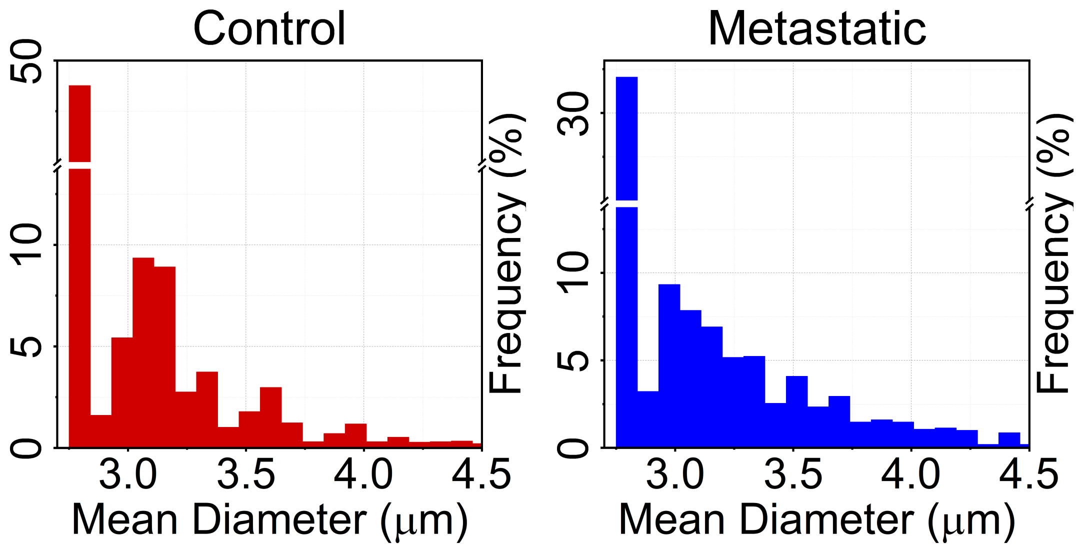

Fig. S1: Histogram distribution of the diameter for the smaller blood vessels obtained from Frangi's segmentation for control and metastatic specimens.

To support X-ray phase-contrast CT scan results, conventional histology Masson's trichrome staining has been performed on both the control and the metastatic specimen. The images, with the corresponding best matched X-ray phase-contrast CT slice, are reported in Fig.S2. The histological slice of the control

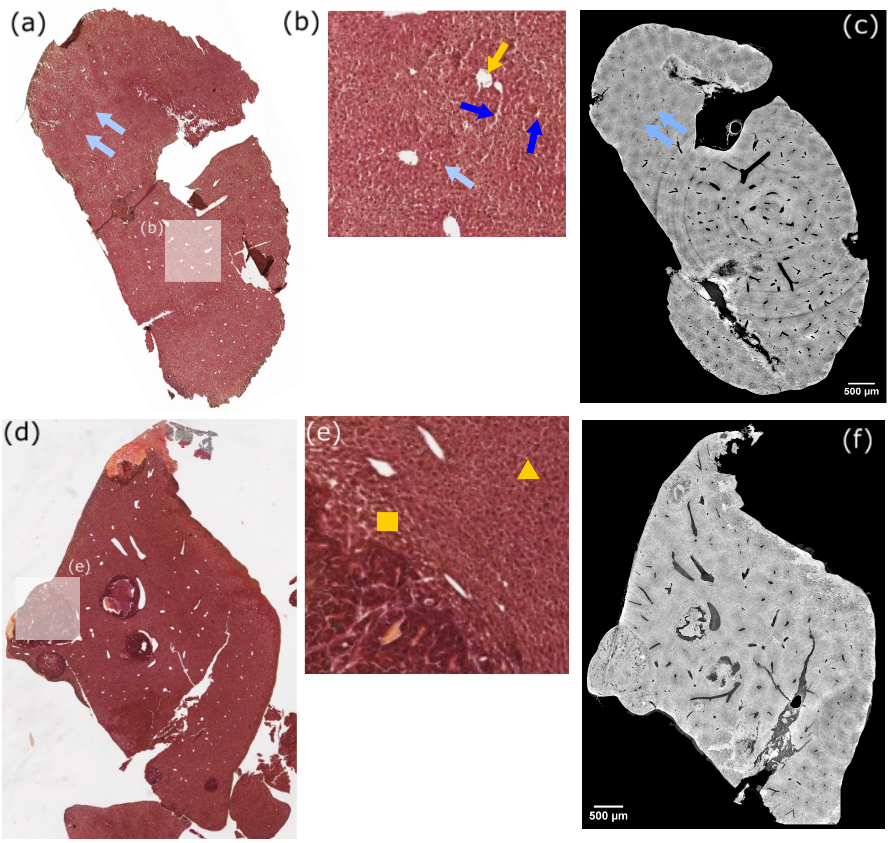

Fig. S2: Panels (a) to (c) show for the control specimen a low-resolution histology slice, a zoomed-in view of the region in the white rectangle, and the best-matched X-ray CT slice. Yellow and blue arrows point to large and small vessels, respectively. Light blue arrows point in the histological and X-ray CT slice to higher-density regions, already commented on in the main paper. Panels (d) to (f) show the same for the metastatic specimen. Yellow triangle and square symbols indicate the tissue identified as tissue 2 in the main paper and a region with a high density of blood vessels, respectively.

specimen shown in S2(a) supports the tissue appearance observed in the CT images as well as the presence of the patchy-like appearance of liver lobule, even if less marked compared to the X-ray image (light blue arrows in panels (a), (c) and the inset in panel (b)). Furthermore, the homogeneous appearance of the tissue is confirmed, along with the identification of large vessels consistent with either a central vein or a portal triad (depicted as yellow arrow in panel(b)) surrounded by a dense network of smaller vessels indicative with sinusoids indicated by the blue arrow in S2(b). Several large metastatic nodules can be observed in the tumoral specimen reported inS2(d). Remarkably, a closer examination around a metastatic nodule (panel(e)) shows both the compact tissue with a lower density of capillaries (yellow triangle) and their increased presence at the edge of the nodule (yellow square) as observed through X-ray phase contrast tomography. Finally, it is worth noting between the histology and CT scans for both specimens, as illustrated in panels (c) and (f), which confirm the accurate identification of all X-ray features.

While the X-ray results are limited to one sample/group, i.e. control and metastatic, additional histological slices from different murine liver samples have been analyzed to validate the results as shown in S3. Specifically, H&E slices from four additional control mice and four metastatic mice have been reported. The sinusoid network is clearly visible around the larger vessels in the control specimens. On the other hand, this vascular architecture is largely lost in the presence of metastatic lesions, confirming the X-ray findings.

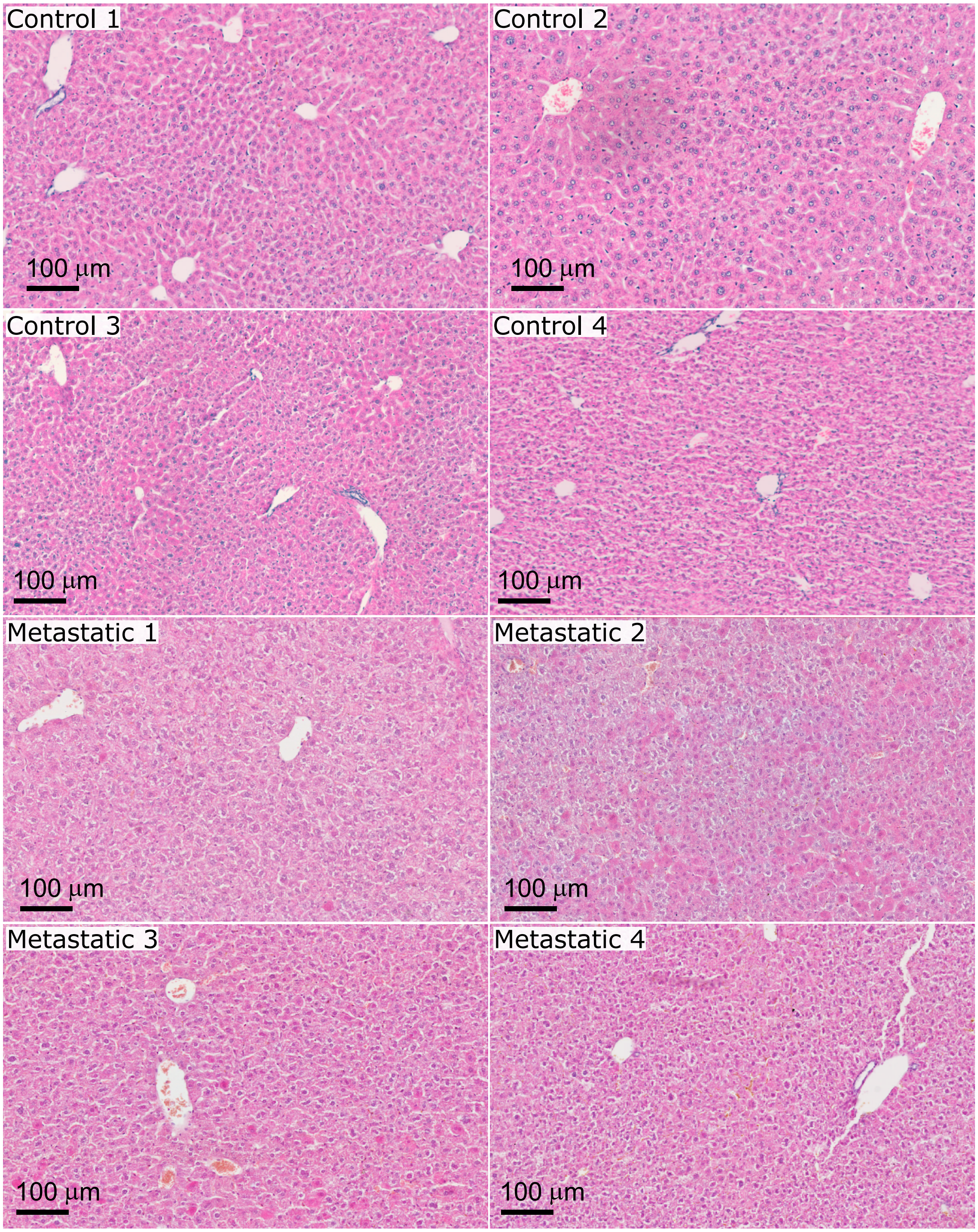

Fig. S3: Representative H&E images of control and metastatic livers from different mice.

Additional CD31 slices from all the available specimens are shown in S4. In the control samples, a dense network of sinusoids is visible, whereas in the metastatic cases, it is notably reduced.

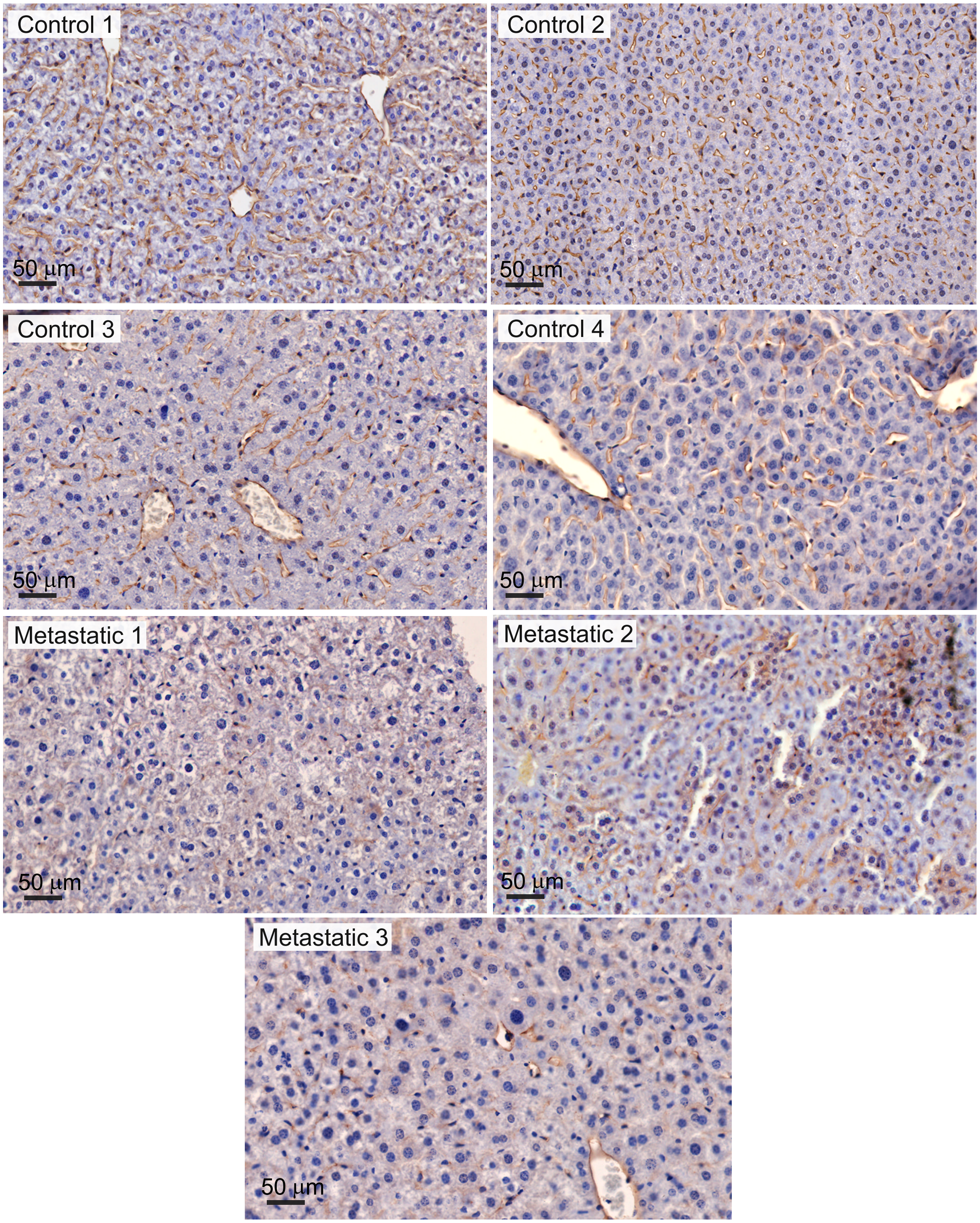

Fig. S4: Representative CD31 IHC images of control and metastatic livers from different mice.

Radial vascular volume fraction was calculated in shells of increasing size around the metastasis, as green areas in S5 overlapped with a tomographic section. For each shell, the vascular volume fraction, i.e. the ratio between the volume of the segmented blood vessels (in red) and the shell, is calculated and plotted against the major axis of the best-fit ellipsoid.

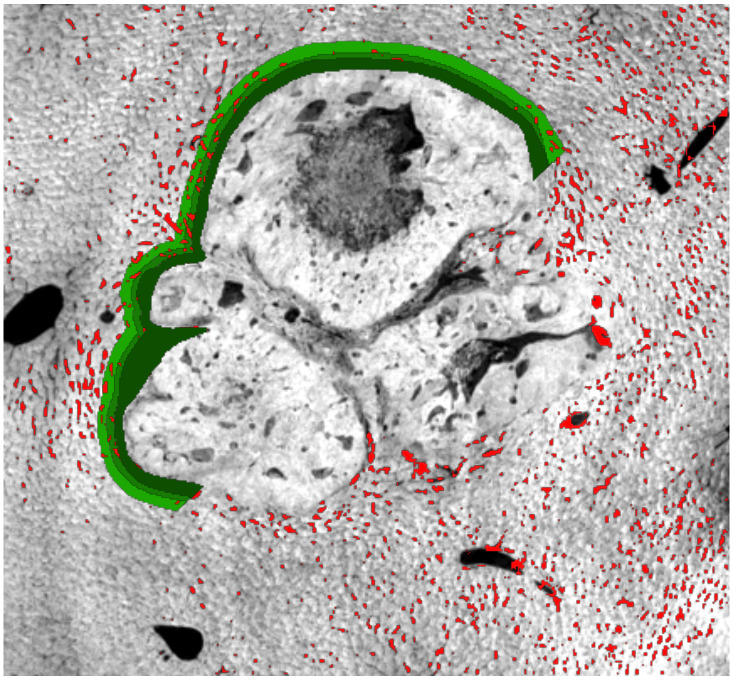

Fig. S5: Schematic illustration of the radial vascular volume fraction calculation.
